# Crystal structure of poly[[aqua­(μ-2,3-di­hydro­thieno[3,4-*b*][1,4]dioxine-5,7-di­carboxyl­ato-κ^2^ *O* ^5^:*O* ^7^)[μ-di(pyridin-4-yl)sulfane-κ^2^ *N*:*N*′]zinc] 0.26-hydrate]

**DOI:** 10.1107/S2056989017002031

**Published:** 2017-02-14

**Authors:** Wen-Liang Wu, Bing Hu

**Affiliations:** a State Key Laboratory of Structural Chemistry, Fujian Institute of Research on the Structure of Matter, Chinese Academy of Sciences, Fuzhou, Fujian 350002, People’s Republic of China

**Keywords:** crystal structure, hydrogen bond, zinc, trigonal–bipyramidal coordination environment

## Abstract

The crystal structure of the title polymer, {[Zn(C_8_H_4_O_6_S)(C_10_H_8_N_2_S)(H_2_O)]·0.26H_2_O}_*n*_, is characterized by a layered arrangement parallel to the *ab* plane. The zinc cation is five-coordinated in a slightly distorted trigonal–bipyramidal coordination environment defined by two pyridine ligands, two carboxyl­ate groups of two thio­phene di­carboxyl­ate ligands, and by one water mol­ecule. The ethyl­ene bridge in the dioxine ligand is disordered over two sets of sites [occupancy ratio 0.624 (9):0.376 (9)]. Several hydrogen-bonding inter­actions of the types O—H⋯O, C—H⋯O, C—H⋯S and C—H⋯N ensure the cohesion within the crystal structure.

## Abstract 2

The zinc cation in the structure has a N_2_O_3_ coordination set, arranged in a trigonal–bipyramidal configuration. The bridging mode of the organic ligands leads to the formation of a polymeric layer structure parallel to the *ab* plane.

## Chemical context   

Complexes constructed by metal ions and organic ligands are of continuous inter­est due to the vast diversity and feasible tailorability of their structures and functions compared with purely inorganic compounds (Zhang *et al.*, 2015 ▸).
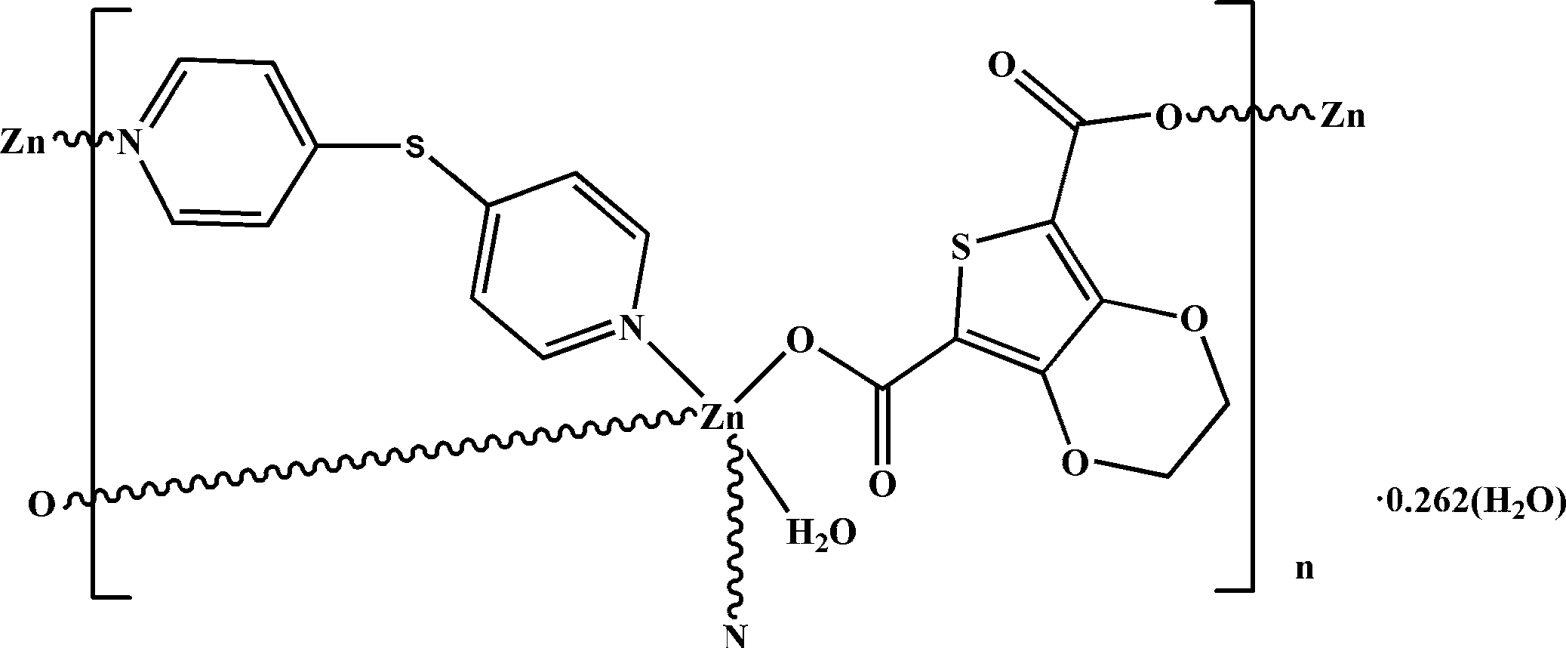


The incorporation of both carb­oxy­lic and pyridine ligands can lead to a variety of structures (Schoedel *et al.*, 2016 ▸). Complexes based on thio­phene derivatives with carb­oxy­lic acid functionalities are of some inter­est as anti­cancer agents (Chen *et al.*, 1998 ▸, 1999 ▸; Guo *et al.*, 2009 ▸). In this context, we report here on synthesis and crystal structure of the title compound, [Zn(C_8_H_4_O_6_S)(C_10_H_8_N_2_S)(H_2_O)]·0.26H_2_O, (1).

## Structural commentary   

**Figure 1:**
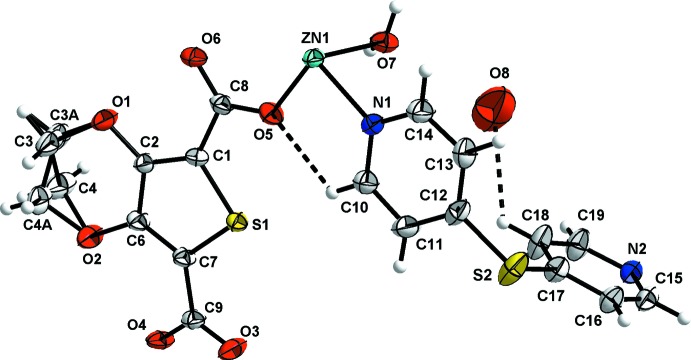
The asymmetric unit of (1), with displacement ellipsoids drawn at the 50% probability level. Hydrogen bonding is indicated by dashed lines.

In the crystal structure of (1), the zinc ion is coordinated by four organic ligands and one water mol­ecule, giving rise to a slightly distorted trigonal–bipyramidal coordination environment. Two nitro­gen atoms are delivered by two symmetry-related pyridine ligands, two oxygen atoms of two carboxyl groups stem from two symmetry-related thio­phene carboxyl­ate ligands, and one O atom from the aqua ligand (Fig. 1 ▸). In the trigonal bipyramid, the axial angle O7—Zn1—N2 is 171.31 (6)°. The Zn^II^ ion is co-planar with the O5—N1—O4 equatorial plane, with the deviation of the Zn atom from this plane being 0.0034 (3) Å. The equatorial Zn1—N1 bond length is 2.1131 (18) Å, while the axial Zn1—N2 bond is longer, 2.2107 (18) Å. Similarly, the two equatorial Zn1—O (O4, O5) bond lengths, ranging from 1.9835 (15) to 2.0285 (15) Å, are shorter than the axial Zn1—O7 bond of 2.1375 (17) Å. These are typical values, numerical details of which are given in Table 1 ▸.

## Supra­molecular features   

**Figure 2:**
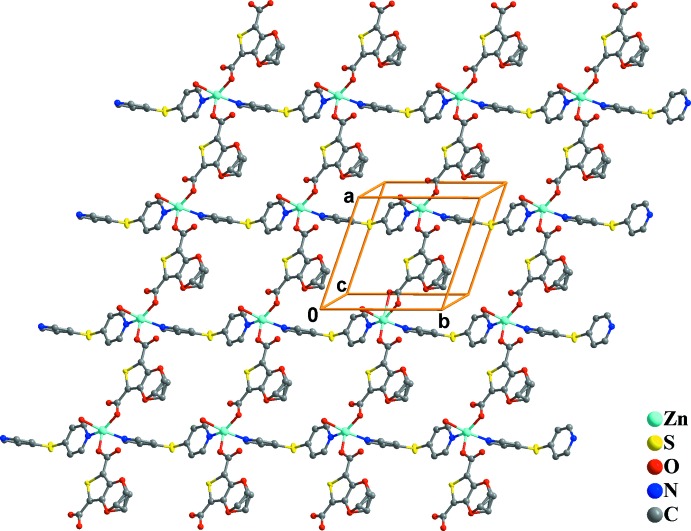
The polymeric layer in the crystal structure of (1), extending along the *ab* plane (H atoms have been omitted for clarity).

The bridging coordinating mode of the organic ligands leads to the formation of polymeric layers parallel to the *ab* plane (Fig. 2 ▸).

**Figure 3:**
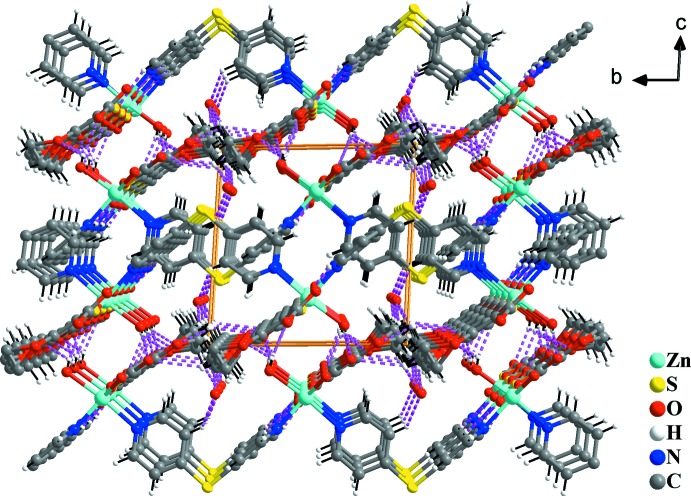
Part of the crystal structure of (1), showing the network formed by inter­molecular C—H⋯O, O—H⋯O, C—H⋯S and C—H⋯N hydrogen bonds (shown as dashed lines).

There are several types of hydrogen bonds in the structure. One intra­molecular hydrogen bond is present and extends from a (pyridine)C—H group (C10—H10*A*) to the coordinating O5 atom of the carboxyl group. Another (pyridine)C—H group (C18—H18*A*) is hydrogen-bonded to the disordered O8 atom of the lattice water mol­ecule. Three O—H⋯O inter­actions are present between the coordinating water mol­ecule to either the carboxyl group oxygen atoms or the dioxine oxygen atom in the thio­phene derivative with *D*⋯*A* distances ranging between 2.733 (2) and 3.123 (2) Å and corresponding O—H⋯O angles of 135 (2) and 159 (2)°. Numerous other C—H⋯O inter­actions are present between the disordered dioxine C—H groups and a carboxyl O atom (O6) or the lattice water atom O8. Other C—H⋯O inter­actions involve pyridyl C—H groups and the carboxyl O3 atom. In addition, one C—H⋯S inter­action and one C—H⋯N inter­action are found between pyridyl C—H groups and the sulfane S1 atom or the pyridyl N1 atom (Fig. 3 ▸). It is expected that other extensive hydrogen bonds are formed with the lattice water mol­ecules as the donor group and the coordinating water mol­ecules or carbonyl O atoms from the layers as acceptors (O8⋯O distances in the range 2.87–3.13 Å). However, since the H atoms of the disordered O8 atom were not modelled, a definite statement cannot be made. Numerical details of the hydrogen bonding are given in Table 2 ▸.

## Database survey   

Some complexes based on tddc^2−^ (H_2_ttdc is 2,3-di­hydro­thieno[3,4-*b*][1,4]dioxine-5,7-di­carb­oxy­lic acid) (Guo *et al.*, 2009 ▸) or di(pyridin-4-yl)sulfane (Liu *et al.*, 2015 ▸; Han *et al.*, 2015 ▸) have been reported, but a complex incorporating both ligands was not found.

## Synthesis and crystallization   

2,3-Di­hydro­thieno[3,4-*b*][1,4]dioxine-5,7-di­carb­oxy­lic acid (H_2_ttdc) was prepared as reported (Zhang *et al.*, 2011 ▸), and di(pyridin-4-yl)sulfane was formed *in situ* from the reactant 4,4′-di­thiodi­pyridine in the synthesis. A mixture of zinc nitrate (0.06 g, 0.21 mmol), H_2_ttdc (0.02 g, 0.10 mmol), 4,4′-di­thiodi­pyridine (0.02 g, 0.10 mmol), 5 ml di­methyl­formamide and 3 ml water was mixed and heated at 353 K for 3 days. After cooling, 0.17 g light-yellow crystals were collected in a yield of 32%.

## Refinement   

Crystal data, data collection and structure refinement details are summarized in Table 3 ▸. H atoms attached to carbon were positioned geometrically and constrained to ride on their parent atoms, with *U*_iso_(H) = 1.2*U*_eq_(C). The H atoms of the coordinating water mol­ecule were located in a difference map and restrained to have comparable bond lengths using DFIX and DANG commands to keep their geometries reasonable; *U*_iso_(H) values were set to 1.5*U*_eq_(O). The hydrogen atoms of the disordered lattice water mol­ecule [occupancy 0.262 (10)] could not be retrieved from difference maps and thus were not part of the model. Two carbon atoms of the dioxine moiety are disordered over two sets of sites and were refined in two parts (C3–C4/C3*A*–C4*A*) with a refined occupancy ratio of 0.624 (9)/0.376 (9). Soft restraints (DFIX, SIMU, SADI) were applied on the disordered atoms to keep their geometries and atomic displacement parameters reasonable.

## Supplementary Material

Crystal structure: contains datablock(s) I. DOI: 10.1107/S2056989017002031/wm5363sup1.cif

Structure factors: contains datablock(s) I. DOI: 10.1107/S2056989017002031/wm5363Isup2.hkl

CCDC reference: 1528425

Additional supporting information:  crystallographic information; 3D view; checkCIF report

## Figures and Tables

**Table 1:** Selected geometric parameters (Å, °)

Zn1—O5	1.9835 (15)	Zn1—O7	2.1375 (17)
Zn1—O4^i^	2.0285 (15)	Zn1—N2^ii^	2.2107 (18)
Zn1—N1	2.1131 (18)		
			
O5—Zn1—O4^i^	117.56 (6)	N1—Zn1—O7	85.43 (7)
O5—Zn1—N1	95.66 (7)	O5—Zn1—N2^ii^	95.24 (7)
O4^i^—Zn1—N1	146.78 (7)	O4^i^—Zn1—N2^ii^	85.85 (7)
O5—Zn1—O7	93.06 (6)	N1—Zn1—N2^ii^	91.17 (7)
O4^i^—Zn1—O7	92.61 (6)	O7—Zn1—N2^ii^	171.31 (6)

Symmetry codes: (i) 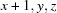; (ii) 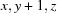.

**Table 2:** Hydrogen-bond geometry (Å, °)

*D*—H⋯*A*	*D*—H	H⋯*A*	*D*⋯*A*	*D*—H⋯*A*
O7—H7*A*⋯O2^iii^	0.83 (1)	2.48 (2)	3.123 (2)	135 (2)
O7—H7*A*⋯O4^iii^	0.83 (1)	2.04 (2)	2.746 (2)	143 (2)
O7—H7*B*⋯O6^iv^	0.83 (1)	1.94 (1)	2.733 (2)	159 (2)
C3—H3*A*⋯O6^v^	0.97	2.66	3.275 (9)	122
C4—H4*A*⋯O8^iii^	0.97	2.27	3.015 (17)	133
C3*A*—H3*D*⋯O6^v^	0.97	2.60	3.473 (19)	150
C4*A*—H4*C*⋯O8^vi^	0.97	1.93	2.566 (16)	121
C10—H10*A*⋯O5	0.93	2.50	3.079 (3)	121
C14—H14*A*⋯O3^i^	0.93	2.51	3.213 (3)	133
C15—H15*A*⋯S1^vii^	0.93	3.01	3.768 (2)	140
C15—H15*A*⋯O3^vii^	0.93	2.57	3.096 (3)	116
C15—H15*A*⋯N1^viii^	0.93	2.67	3.227 (3)	119
C18—H18*A*⋯O8	0.93	2.58	3.190 (13)	123

Symmetry codes: (i) 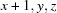; (iii) 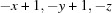; (iv) 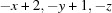; (v) 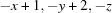; (vi) 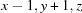; (vii) 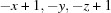; (viii) 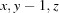.

**Table 3:** Experimental details

Crystal data	
Chemical formula	[Zn(C_8_H_4_O_6_S)(C_10_H_8_N_2_S)(H_2_O)]·0.26H_2_O
*M*_r_	504.57
Crystal system, space group	Triclinic, *P* 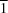
Temperature (K)	295
*a*, *b*, *c* (Å)	10.0052 (6), 10.2173 (5), 10.6694 (5)
α, β, γ (°)	87.515 (4), 68.625 (5), 73.988 (5)
*V* (Å^3^)	974.27 (10)
*Z*	2
Radiation type	Mo *K*α
μ (mm^−1^)	1.52
Crystal size (mm)	0.32 × 0.25 × 0.20

Data collection	
Diffractometer	Rigaku SuperNova, single source at offset, EosS2
Absorption correction	Multi-scan (*CrysAlis PRO*; Rigaku OD, 2015 ▸)
*T*_min_, *T*_max_	0.784, 1.000
No. of measured, independent and observed [*I* > 2σ(*I*)] reflections	10924, 3925, 3549
*R*_int_	0.021
(sin θ/λ)_max_ (Å^−1^)	0.659

Refinement	
*R*[*F* ^2^ > 2σ(*F* ^2^)], *wR*(*F* ^2^), *S*	0.030, 0.069, 1.01
No. of reflections	3925
No. of parameters	306
No. of restraints	47
H-atom treatment	H atoms treated by a mixture of independent and constrained refinement
Δρ_max_, Δρ_min_ (e Å^−3^)	0.58, −0.39

Computer programs: *CrysAlis PRO* (Rigaku OD, 2015 ▸), *SHELXT2014* (Sheldrick, 2015*a* ▸), *SHELXL2014* (Sheldrick, 2015*b* ▸), *DIAMOND* (Brandenburg & Putz, 2004 ▸) and *publCIF* (Westrip, 2010 ▸).

## supplementary crystallographic information

### Crystal data

**Table:**

[Zn(C_8_H_4_O_6_S)(C_10_H_8_N_2_S)(H_2_O)]·0.26H_2_O	*Z* = 2
*M**_r_* = 504.57	*F*(000) = 512.7
Triclinic, *P*1	*D*_x_ = 1.718 Mg m^−^^3^
*a* = 10.0052 (6) Å	Mo *K*α radiation, λ = 0.71073 Å
*b* = 10.2173 (5) Å	Cell parameters from 6096 reflections
*c* = 10.6694 (5) Å	θ = 4.1–27.2°
α = 87.515 (4)°	µ = 1.52 mm^−^^1^
β = 68.625 (5)°	*T* = 295 K
γ = 73.988 (5)°	Block, yellow
*V* = 974.27 (10) Å^3^	0.32 × 0.25 × 0.20 mm

### Data collection

**Table:**

Rigaku SuperNova, single source at offset, EosS2 diffractometer	3925 independent reflections
Radiation source: micro-focus sealed X-ray tube	3549 reflections with *I* > 2σ(*I*)
Detector resolution: 8.0584 pixels mm^-1^	*R*_int_ = 0.021
ω scans	θ_max_ = 27.9°, θ_min_ = 3.5°
Absorption correction: multi-scan (*CrysAlis PRO*; Rigaku OD, 2015)	*h* = −12→12
*T*_min_ = 0.784, *T*_max_ = 1.000	*k* = −13→13
10924 measured reflections	*l* = −14→13

### Refinement

**Table:**

Refinement on *F*^2^	47 restraints
Least-squares matrix: full	Hydrogen site location: mixed
*R*[*F*^2^ > 2σ(*F*^2^)] = 0.030	H atoms treated by a mixture of independent and constrained refinement
*wR*(*F*^2^) = 0.069	*w* = 1/[σ^2^(*F*_o_^2^) + (0.0267*P*)^2^ + 0.8*P*] where *P* = (*F*_o_^2^ + 2*F*_c_^2^)/3
*S* = 1.01	(Δ/σ)_max_ < 0.001
3925 reflections	Δρ_max_ = 0.58 e Å^−^^3^
306 parameters	Δρ_min_ = −0.39 e Å^−^^3^

### Special details

**Table:**

Geometry. All esds (except the esd in the dihedral angle between two l.s. planes) are estimated using the full covariance matrix. The cell esds are taken into account individually in the estimation of esds in distances, angles and torsion angles; correlations between esds in cell parameters are only used when they are defined by crystal symmetry. An approximate (isotropic) treatment of cell esds is used for estimating esds involving l.s. planes.

### Fractional atomic coordinates and isotropic or equivalent isotropic displacement parameters (Å^2^)

**Table:**

	*x*	*y*	*z*	*U*_iso_*/*U*_eq_	Occ. (<1)
Zn1	0.87280 (3)	0.50627 (3)	0.23783 (3)	0.02570 (9)	
S1	0.40744 (6)	0.53672 (5)	0.19475 (5)	0.02461 (13)	
S2	0.66583 (9)	0.04878 (7)	0.67347 (6)	0.04739 (19)	
O1	0.46527 (18)	0.85023 (18)	−0.01437 (18)	0.0414 (4)	
O2	0.17934 (19)	0.80589 (19)	0.0274 (2)	0.0467 (5)	
O3	0.11429 (19)	0.47305 (18)	0.27948 (18)	0.0425 (4)	
O4	0.03227 (16)	0.59934 (17)	0.13592 (15)	0.0315 (4)	
O5	0.69482 (17)	0.54177 (17)	0.18673 (16)	0.0341 (4)	
O6	0.72194 (17)	0.71896 (18)	0.05940 (18)	0.0417 (4)	
O7	0.98637 (17)	0.32423 (18)	0.10848 (16)	0.0331 (4)	
H7A	0.949 (2)	0.331 (3)	0.0497 (19)	0.050*	
H7B	1.0774 (12)	0.315 (3)	0.075 (2)	0.050*	
N1	0.8030 (2)	0.37482 (18)	0.39130 (18)	0.0271 (4)	
N2	0.7799 (2)	−0.31732 (19)	0.38683 (18)	0.0293 (4)	
C1	0.5018 (2)	0.6525 (2)	0.1156 (2)	0.0234 (4)	
C2	0.4201 (2)	0.7464 (2)	0.0563 (2)	0.0260 (5)	
C3	0.3432 (9)	0.9506 (9)	−0.0350 (10)	0.050 (2)	0.624 (9)
H3A	0.3823	1.0115	−0.1018	0.060*	0.624 (9)
H3B	0.2798	1.0046	0.0487	0.060*	0.624 (9)
C4	0.2518 (6)	0.8826 (6)	−0.0820 (6)	0.0520 (16)	0.624 (9)
H4A	0.1773	0.9509	−0.1056	0.062*	0.624 (9)
H4B	0.3160	0.8218	−0.1611	0.062*	0.624 (9)
C3A	0.3637 (11)	0.9273 (19)	−0.0762 (13)	0.049 (4)	0.376 (9)
H3C	0.3861	0.8817	−0.1623	0.059*	0.376 (9)
H3D	0.3792	1.0171	−0.0932	0.059*	0.376 (9)
C4A	0.2024 (9)	0.9430 (7)	0.0105 (12)	0.055 (3)	0.376 (9)
H4C	0.1787	0.9870	0.0977	0.066*	0.376 (9)
H4D	0.1377	0.9989	−0.0322	0.066*	0.376 (9)
C6	0.2801 (2)	0.7233 (2)	0.0760 (2)	0.0275 (5)	
C7	0.2570 (2)	0.6134 (2)	0.1493 (2)	0.0246 (4)	
C8	0.6522 (2)	0.6389 (2)	0.1185 (2)	0.0270 (5)	
C9	0.1274 (2)	0.5569 (2)	0.1920 (2)	0.0271 (5)	
C10	0.6685 (3)	0.3545 (3)	0.4234 (3)	0.0364 (5)	
H10A	0.6029	0.4076	0.3856	0.044*	
C11	0.6230 (3)	0.2585 (3)	0.5098 (3)	0.0419 (6)	
H11A	0.5276	0.2488	0.5314	0.050*	
C12	0.7185 (3)	0.1776 (2)	0.5640 (2)	0.0333 (5)	
C13	0.8558 (3)	0.1991 (3)	0.5352 (3)	0.0445 (6)	
H13A	0.9224	0.1476	0.5728	0.053*	
C14	0.8924 (3)	0.2991 (3)	0.4487 (3)	0.0452 (7)	
H14A	0.9852	0.3141	0.4299	0.054*	
C15	0.7464 (3)	−0.3252 (2)	0.5191 (2)	0.0332 (5)	
H15A	0.7490	−0.4104	0.5540	0.040*	
C16	0.7082 (3)	−0.2146 (2)	0.6076 (2)	0.0347 (5)	
H16A	0.6836	−0.2252	0.6995	0.042*	
C17	0.7074 (3)	−0.0877 (2)	0.5566 (2)	0.0331 (5)	
C18	0.7401 (3)	−0.0775 (3)	0.4195 (3)	0.0446 (7)	
H18A	0.7383	0.0065	0.3820	0.054*	
C19	0.7751 (3)	−0.1935 (2)	0.3391 (2)	0.0404 (6)	
H19A	0.7966	−0.1852	0.2472	0.049*	
O8	0.9744 (16)	0.0639 (13)	0.2164 (12)	0.126 (7)	0.262 (10)

### Atomic displacement parameters (Å^2^)

**Table:**

	*U*^11^	*U*^22^	*U*^33^	*U*^12^	*U*^13^	*U*^23^
Zn1	0.02134 (13)	0.02812 (15)	0.03265 (15)	−0.01234 (10)	−0.01248 (10)	0.00955 (10)
S1	0.0224 (3)	0.0256 (3)	0.0299 (3)	−0.0091 (2)	−0.0128 (2)	0.0050 (2)
S2	0.0788 (5)	0.0330 (3)	0.0288 (3)	−0.0272 (3)	−0.0098 (3)	0.0049 (3)
O1	0.0354 (9)	0.0444 (10)	0.0579 (11)	−0.0242 (8)	−0.0256 (8)	0.0275 (9)
O2	0.0373 (10)	0.0506 (11)	0.0721 (13)	−0.0245 (9)	−0.0374 (9)	0.0357 (10)
O3	0.0427 (10)	0.0491 (11)	0.0498 (11)	−0.0300 (9)	−0.0231 (8)	0.0265 (9)
O4	0.0234 (8)	0.0453 (10)	0.0338 (8)	−0.0185 (7)	−0.0139 (7)	0.0095 (7)
O5	0.0248 (8)	0.0393 (10)	0.0456 (10)	−0.0101 (7)	−0.0213 (7)	0.0103 (8)
O6	0.0235 (8)	0.0425 (10)	0.0624 (12)	−0.0158 (8)	−0.0162 (8)	0.0160 (9)
O7	0.0253 (8)	0.0427 (10)	0.0341 (9)	−0.0126 (8)	−0.0118 (7)	0.0031 (7)
N1	0.0269 (9)	0.0250 (10)	0.0312 (10)	−0.0107 (8)	−0.0105 (8)	0.0052 (8)
N2	0.0330 (10)	0.0268 (10)	0.0317 (10)	−0.0117 (8)	−0.0139 (8)	0.0063 (8)
C1	0.0190 (10)	0.0273 (11)	0.0252 (11)	−0.0090 (8)	−0.0079 (8)	0.0016 (9)
C2	0.0242 (10)	0.0282 (12)	0.0284 (11)	−0.0122 (9)	−0.0098 (9)	0.0058 (9)
C3	0.050 (4)	0.046 (4)	0.073 (5)	−0.026 (3)	−0.039 (3)	0.035 (4)
C4	0.051 (3)	0.065 (3)	0.061 (3)	−0.032 (3)	−0.036 (3)	0.039 (3)
C3A	0.046 (5)	0.049 (6)	0.061 (7)	−0.020 (4)	−0.027 (5)	0.034 (5)
C4A	0.046 (4)	0.047 (5)	0.083 (6)	−0.016 (4)	−0.038 (4)	0.036 (4)
C6	0.0228 (10)	0.0326 (12)	0.0329 (12)	−0.0108 (9)	−0.0152 (9)	0.0084 (9)
C7	0.0208 (10)	0.0295 (12)	0.0270 (11)	−0.0091 (9)	−0.0112 (8)	0.0032 (9)
C8	0.0198 (10)	0.0305 (12)	0.0317 (12)	−0.0075 (9)	−0.0098 (9)	−0.0006 (9)
C9	0.0236 (11)	0.0298 (12)	0.0308 (12)	−0.0131 (9)	−0.0090 (9)	0.0024 (9)
C10	0.0284 (12)	0.0387 (14)	0.0433 (14)	−0.0120 (10)	−0.0137 (10)	0.0111 (11)
C11	0.0338 (13)	0.0492 (16)	0.0473 (15)	−0.0228 (12)	−0.0132 (11)	0.0147 (12)
C12	0.0457 (14)	0.0235 (12)	0.0271 (12)	−0.0130 (10)	−0.0069 (10)	0.0013 (9)
C13	0.0433 (15)	0.0397 (15)	0.0514 (16)	−0.0093 (12)	−0.0215 (12)	0.0172 (12)
C14	0.0315 (13)	0.0500 (16)	0.0605 (17)	−0.0187 (12)	−0.0208 (12)	0.0225 (13)
C15	0.0398 (13)	0.0285 (12)	0.0360 (13)	−0.0141 (10)	−0.0168 (11)	0.0114 (10)
C16	0.0429 (14)	0.0339 (13)	0.0292 (12)	−0.0141 (11)	−0.0136 (10)	0.0078 (10)
C17	0.0396 (13)	0.0279 (12)	0.0324 (12)	−0.0116 (10)	−0.0126 (10)	0.0036 (9)
C18	0.0727 (19)	0.0260 (13)	0.0367 (14)	−0.0166 (13)	−0.0206 (13)	0.0100 (10)
C19	0.0594 (17)	0.0330 (14)	0.0293 (13)	−0.0135 (12)	−0.0169 (12)	0.0072 (10)
O8	0.138 (13)	0.101 (10)	0.094 (10)	−0.032 (8)	0.007 (8)	0.002 (7)

### Geometric parameters (Å, º)

**Table:**

Zn1—O5	1.9835 (15)	C2—C6	1.424 (3)
Zn1—O4^i^	2.0285 (15)	C3—C4	1.512 (8)
Zn1—N1	2.1131 (18)	C3—H3A	0.9700
Zn1—O7	2.1375 (17)	C3—H3B	0.9700
Zn1—N2^ii^	2.2107 (18)	C4—H4A	0.9700
S1—C1	1.718 (2)	C4—H4B	0.9700
S1—C7	1.721 (2)	C3A—C4A	1.506 (9)
S2—C17	1.768 (2)	C3A—H3C	0.9700
S2—C12	1.781 (2)	C3A—H3D	0.9700
O1—C2	1.358 (3)	C4A—H4C	0.9700
O1—C3	1.441 (5)	C4A—H4D	0.9700
O1—C3A	1.446 (7)	C6—C7	1.364 (3)
O2—C6	1.367 (3)	C7—C9	1.477 (3)
O2—C4	1.456 (4)	C10—C11	1.371 (3)
O2—C4A	1.474 (6)	C10—H10A	0.9300
O3—C9	1.236 (3)	C11—C12	1.363 (3)
O4—C9	1.271 (3)	C11—H11A	0.9300
O4—Zn1^iii^	2.0285 (15)	C12—C13	1.372 (4)
O5—C8	1.275 (3)	C13—C14	1.382 (4)
O6—C8	1.226 (3)	C13—H13A	0.9300
O7—H7A	0.827 (9)	C14—H14A	0.9300
O7—H7B	0.828 (9)	C15—C16	1.380 (3)
N1—C14	1.323 (3)	C15—H15A	0.9300
N1—C10	1.336 (3)	C16—C17	1.383 (3)
N2—C15	1.331 (3)	C16—H16A	0.9300
N2—C19	1.339 (3)	C17—C18	1.384 (3)
N2—Zn1^iv^	2.2107 (18)	C18—C19	1.379 (3)
C1—C2	1.372 (3)	C18—H18A	0.9300
C1—C8	1.484 (3)	C19—H19A	0.9300
			
O5—Zn1—O4^i^	117.56 (6)	H3C—C3A—H3D	107.8
O5—Zn1—N1	95.66 (7)	O2—C4A—C3A	108.1 (11)
O4^i^—Zn1—N1	146.78 (7)	O2—C4A—H4C	110.1
O5—Zn1—O7	93.06 (6)	C3A—C4A—H4C	110.1
O4^i^—Zn1—O7	92.61 (6)	O2—C4A—H4D	110.1
N1—Zn1—O7	85.43 (7)	C3A—C4A—H4D	110.1
O5—Zn1—N2^ii^	95.24 (7)	H4C—C4A—H4D	108.4
O4^i^—Zn1—N2^ii^	85.85 (7)	C7—C6—O2	124.33 (19)
N1—Zn1—N2^ii^	91.17 (7)	C7—C6—C2	113.23 (19)
O7—Zn1—N2^ii^	171.31 (6)	O2—C6—C2	122.4 (2)
C1—S1—C7	92.49 (10)	C6—C7—C9	129.87 (19)
C17—S2—C12	101.52 (11)	C6—C7—S1	110.81 (15)
C2—O1—C3	111.8 (4)	C9—C7—S1	119.32 (17)
C2—O1—C3A	113.7 (7)	O6—C8—O5	126.4 (2)
C6—O2—C4	111.1 (2)	O6—C8—C1	119.5 (2)
C6—O2—C4A	110.1 (3)	O5—C8—C1	114.1 (2)
C9—O4—Zn1^iii^	101.88 (14)	O3—C9—O4	122.8 (2)
C8—O5—Zn1	126.61 (15)	O3—C9—C7	120.0 (2)
Zn1—O7—H7A	106.5 (19)	O4—C9—C7	117.1 (2)
Zn1—O7—H7B	110.6 (19)	N1—C10—C11	122.7 (2)
H7A—O7—H7B	111.7 (16)	N1—C10—H10A	118.7
C14—N1—C10	117.0 (2)	C11—C10—H10A	118.7
C14—N1—Zn1	122.99 (16)	C12—C11—C10	119.6 (2)
C10—N1—Zn1	119.57 (16)	C12—C11—H11A	120.2
C15—N2—C19	116.8 (2)	C10—C11—H11A	120.2
C15—N2—Zn1^iv^	125.22 (15)	C11—C12—C13	118.6 (2)
C19—N2—Zn1^iv^	117.37 (15)	C11—C12—S2	121.1 (2)
C2—C1—C8	129.8 (2)	C13—C12—S2	120.2 (2)
C2—C1—S1	111.10 (15)	C12—C13—C14	118.2 (2)
C8—C1—S1	119.09 (16)	C12—C13—H13A	120.9
O1—C2—C1	124.96 (19)	C14—C13—H13A	120.9
O1—C2—C6	122.68 (19)	N1—C14—C13	123.7 (2)
C1—C2—C6	112.4 (2)	N1—C14—H14A	118.2
O1—C3—C4	110.7 (6)	C13—C14—H14A	118.2
O1—C3—H3A	109.5	N2—C15—C16	124.0 (2)
C4—C3—H3A	109.5	N2—C15—H15A	118.0
O1—C3—H3B	109.5	C16—C15—H15A	118.0
C4—C3—H3B	109.5	C15—C16—C17	118.6 (2)
H3A—C3—H3B	108.1	C15—C16—H16A	120.7
O2—C4—C3	108.1 (6)	C17—C16—H16A	120.7
O2—C4—H4A	110.1	C16—C17—C18	118.0 (2)
C3—C4—H4A	110.1	C16—C17—S2	116.68 (18)
O2—C4—H4B	110.1	C18—C17—S2	125.28 (19)
C3—C4—H4B	110.1	C19—C18—C17	119.2 (2)
H4A—C4—H4B	108.4	C19—C18—H18A	120.4
O1—C3A—C4A	112.5 (8)	C17—C18—H18A	120.4
O1—C3A—H3C	109.1	N2—C19—C18	123.3 (2)
C4A—C3A—H3C	109.1	N2—C19—H19A	118.4
O1—C3A—H3D	109.1	C18—C19—H19A	118.4
C4A—C3A—H3D	109.1		
			
C7—S1—C1—C2	−0.11 (17)	Zn1—O5—C8—C1	−170.36 (13)
C7—S1—C1—C8	−179.57 (17)	C2—C1—C8—O6	−1.2 (3)
C3—O1—C2—C1	−165.4 (5)	S1—C1—C8—O6	178.18 (17)
C3A—O1—C2—C1	174.9 (8)	C2—C1—C8—O5	177.9 (2)
C3—O1—C2—C6	14.9 (5)	S1—C1—C8—O5	−2.7 (3)
C3A—O1—C2—C6	−4.7 (8)	Zn1^iii^—O4—C9—O3	3.9 (3)
C8—C1—C2—O1	−0.2 (4)	Zn1^iii^—O4—C9—C7	−175.43 (15)
S1—C1—C2—O1	−179.60 (17)	C6—C7—C9—O3	−166.0 (2)
C8—C1—C2—C6	179.5 (2)	S1—C7—C9—O3	13.6 (3)
S1—C1—C2—C6	0.1 (2)	C6—C7—C9—O4	13.4 (3)
C2—O1—C3—C4	−46.5 (8)	S1—C7—C9—O4	−167.03 (16)
C3A—O1—C3—C4	53 (3)	C14—N1—C10—C11	−1.1 (4)
C6—O2—C4—C3	−50.6 (6)	Zn1—N1—C10—C11	171.90 (19)
C4A—O2—C4—C3	46.4 (6)	N1—C10—C11—C12	−1.5 (4)
O1—C3—C4—O2	66.3 (9)	C10—C11—C12—C13	3.1 (4)
C2—O1—C3A—C4A	36.5 (17)	C10—C11—C12—S2	−178.43 (19)
C3—O1—C3A—C4A	−51 (2)	C17—S2—C12—C11	83.8 (2)
C6—O2—C4A—C3A	54.2 (9)	C17—S2—C12—C13	−97.7 (2)
C4—O2—C4A—C3A	−45.6 (7)	C11—C12—C13—C14	−2.1 (4)
O1—C3A—C4A—O2	−62.5 (17)	S2—C12—C13—C14	179.4 (2)
C4—O2—C6—C7	−160.5 (4)	C10—N1—C14—C13	2.1 (4)
C4A—O2—C6—C7	154.0 (5)	Zn1—N1—C14—C13	−170.6 (2)
C4—O2—C6—C2	20.7 (4)	C12—C13—C14—N1	−0.5 (4)
C4A—O2—C6—C2	−24.9 (6)	C19—N2—C15—C16	−0.1 (4)
O1—C2—C6—C7	179.7 (2)	Zn1^iv^—N2—C15—C16	170.64 (18)
C1—C2—C6—C7	0.0 (3)	N2—C15—C16—C17	−1.5 (4)
O1—C2—C6—O2	−1.4 (3)	C15—C16—C17—C18	2.2 (4)
C1—C2—C6—O2	179.0 (2)	C15—C16—C17—S2	−177.25 (19)
O2—C6—C7—C9	0.6 (4)	C12—S2—C17—C16	167.2 (2)
C2—C6—C7—C9	179.5 (2)	C12—S2—C17—C18	−12.2 (3)
O2—C6—C7—S1	−179.02 (18)	C16—C17—C18—C19	−1.4 (4)
C2—C6—C7—S1	−0.1 (2)	S2—C17—C18—C19	178.0 (2)
C1—S1—C7—C6	0.11 (17)	C15—N2—C19—C18	1.0 (4)
C1—S1—C7—C9	−179.53 (17)	Zn1^iv^—N2—C19—C18	−170.5 (2)
Zn1—O5—C8—O6	8.7 (3)	C17—C18—C19—N2	−0.2 (4)

Symmetry codes: (i) *x*+1, *y*, *z*; (ii) *x*, *y*+1, *z*; (iii) *x*−1, *y*, *z*; (iv) *x*, *y*−1, *z*.

### Hydrogen-bond geometry (Å, º)

**Table:**

*D*—H···*A*	*D*—H	H···*A*	*D*···*A*	*D*—H···*A*
O7—H7*A*···O2^v^	0.83 (1)	2.48 (2)	3.123 (2)	135 (2)
O7—H7*A*···O4^v^	0.83 (1)	2.04 (2)	2.746 (2)	143 (2)
O7—H7*B*···O6^vi^	0.83 (1)	1.94 (1)	2.733 (2)	159 (2)
C3—H3*A*···O6^vii^	0.97	2.66	3.275 (9)	122
C4—H4*A*···O8^v^	0.97	2.27	3.015 (17)	133
C3*A*—H3*D*···O6^vii^	0.97	2.60	3.473 (19)	150
C4*A*—H4*C*···O8^viii^	0.97	1.93	2.566 (16)	121
C10—H10*A*···O5	0.93	2.50	3.079 (3)	121
C14—H14*A*···O3^i^	0.93	2.51	3.213 (3)	133
C15—H15*A*···S1^ix^	0.93	3.01	3.768 (2)	140
C15—H15*A*···O3^ix^	0.93	2.57	3.096 (3)	116
C15—H15*A*···N1^iv^	0.93	2.67	3.227 (3)	119
C18—H18*A*···O8	0.93	2.58	3.190 (13)	123

Symmetry codes: (i) *x*+1, *y*, *z*; (iv) *x*, *y*−1, *z*; (v) −*x*+1, −*y*+1, −*z*; (vi) −*x*+2, −*y*+1, −*z*; (vii) −*x*+1, −*y*+2, −*z*; (viii) *x*−1, *y*+1, *z*; (ix) −*x*+1, −*y*, −*z*+1.
